# Associations of education with 30 year life course blood pressure trajectories: Framingham Offspring Study

**DOI:** 10.1186/1471-2458-11-139

**Published:** 2011-02-28

**Authors:** Eric B Loucks, Michal Abrahamowicz, Yongling Xiao, John W Lynch

**Affiliations:** 1 Department of Community Health, Epidemiology Section, Center for Population Health and Clinical Epidemiology, Brown University, Providence, Rhode Island, USA; 2 Department of Epidemiology, Biostatistics and Occupational Health, McGill University, Montreal, Quebec, Canada; 3 School of Health Sciences, University of South Australia, Adelaide, South Australia, Australia; 4 Department of Social Medicine, University of Bristol, Bristol, UK

## Abstract

**Background:**

Education is inversely associated with cardiovascular disease incidence in developed countries. Blood pressure may be an explanatory biological mechanism. However few studies have investigated educational gradients in longitudinal blood pressure trajectories, particularly over substantial proportions of the life course. Study objectives were to determine whether low education was associated with increased blood pressure from multiple longitudinal assessments over 30 years. Furthermore, we aimed to separate antecedent effects of education, and other related factors, that might have caused baseline differences in blood pressure, from potential long-term effects of education on post-baseline blood pressure changes.

**Methods:**

The study examined 3890 participants of the Framingham Offspring Study (mean age 36.7 years, 52.0% females at baseline) from 1971 through 2001 at up to 7 separate examinations using multivariable mixed linear models.

**Results:**

Mixed linear models demonstrated that mean systolic blood pressure (SBP) over 30 years was higher for participants with ≤12 vs. ≥17 years education after adjusting for age (3.26 mmHg, 95% CI: 1.46, 5.05 in females, 2.26 mmHg, 95% CI: 0.87, 3.66 in males). Further adjustment for conventional covariates (antihypertensive medication, smoking, body mass index and alcohol) reduced differences in females and males (2.86, 95% CI: 1.13, 4.59, and 1.25, 95% CI: -0.16, 2.66 mmHg, respectively). Additional analyses adjusted for baseline SBP, to evaluate if there may be educational contributions to post-baseline SBP. In analyses adjusted for age and baseline SBP, females with ≤12 years education had 2.69 (95% CI: 1.09, 4.30) mmHg higher SBP over follow-up compared with ≥17 years education. Further adjustment for aforementioned covariates slightly reduced effect strength (2.53 mmHg, 95% CI: 0.93, 4.14). Associations were weaker in males, where those with ≤12 years education had 1.20 (95% CI: -0.07, 2.46) mmHg higher SBP over follow-up compared to males with ≥17 years of education, after adjustment for age and baseline blood pressure; effects were substantially reduced after adjusting for aforementioned covariates (0.34 mmHg, 95% CI: -0.90, 1.68). Sex-by-education interaction was marginally significant (p = 0.046).

**Conclusion:**

Education was inversely associated with higher systolic blood pressure throughout a 30-year life course span, and associations may be stronger in females than males.

## Background

Education and other measures of socioeconomic position, such as occupation and income, are consistently inversely associated with incidence of cardiovascular disease in developed countries [1,2]. Elevated blood pressure, a major risk factor for cardiovascular disease, has been demonstrated in cross-sectional studies to be associated with low education and lower levels of other SEP measures [3]. Because of the limitations of cross-sectional studies, further investigation of whether educational attainment may be causally related to blood pressure can be achieved through prospective designs that measure longitudinal trajectories of blood pressure. Few studies have investigated longitudinal blood pressure trajectories, especially over a substantial proportion of the life course [4-7]. Furthermore, little is known about the effects of adjusting for potential explanatory/mediating mechanisms such as smoking, alcohol consumption, obesity, or use of antihypertensive medications [4-7].

The objectives of this study were to determine whether low educational attainment was associated with increased blood pressure from multiple longitudinal assessments over 30 years. Furthermore, we aimed to separate 'antecedant' effects of education, and other related factors, that might have caused baseline differences in blood pressure, from potential long-term effects of education on post-baseline changes in blood pressure. Analyses prioritized measures of systolic blood pressure (SBP) over diastolic blood pressure (DBP), as systolic hypertension is substantially more common than diastolic hypertension, and SBP contributes more to the global disease burden attributable to hypertension than DBP [8].

## Methods

### Study sample

The Framingham Heart Study is a community-based, longitudinal, observational cohort study that was initiated in 1948 to prospectively investigate risk factors for coronary heart disease. The Framingham Offspring Study began in 1971 with recruitment of 5124 men and women who were offspring (or offspring's spouses) of the Original Cohort of the Framingham Heart Study. The design and selection criteria of the Framingham Offspring Study have been described elsewhere.[9] Participants were prospectively assessed during 7 examinations between 1971 and 2001. The consecutive examination dates were as follows: 1971-1975; 1979-1982; 1984-1987; 1987-1990; 1991-1995; 1996-1998, and 1998-2001. At each examination visit, participants underwent medical history, physical examination, anthropometry, and laboratory assessment of coronary heart disease risk factors, as previously described.[9] Framingham participants signed informed consent and the study is reviewed annually by the Boston University Medical Center Institutional Review Board.

There were 5124 participants who completed Offspring Examination 1 (in 1971-1975), of which 4989 (97%) agreed for their data to be in the open-access dataset. Of these, 1099 subjects were excluded from the present analyses because of missing education data (primarily among the participants who did not attend exams 2 or 3 when education was assessed) or being <28 years of age (n = 60) when education was assessed. Participants were restricted to those aged ≥28 years at the time education was assessed in order to allow at least 10 years from expected completion of high school (at age 18 years, on average), during which the participants could obtain higher levels of education. Consequently, 3890 subjects were included in the data analyses.

### Education

The participants' own education was measured directly from Framingham Offspring Study participants at Examinations 2 (1979-1982) and 3 (1984-1987). Examination 3 education was used whenever available, otherwise the Examination 2 measure was used. In the original data, education was recorded in 6 categories of completed years of education: 0-4, 5-8, 9-11, 12, 13-16, ≥17 years. For current analyses, the participants' own education was collapsed into 3 groups: ≤12 years (reflecting high school or less), 13-16 years (indicative of some post-secondary education including technical school and college degree) and ≥17 years education (approximating those with more than an undergraduate college degree). This grouping was motivated by both (i) statistical power considerations (to ensure adequate number of participants in each category) and (ii) substantive reasons, whereby the education categories represent educational milestones recognized to influence earnings, occupation type, and socioeconomic position in society.

### Blood Pressure

Each participant rested for at least five minutes before blood pressure measurement. While the participant remained seated, a physician measured SBP and DBP each twice in the left arm with a mercury-column sphygmomanometer, according to a standardized protocol [10]. The average of the two readings was used for analyses.

### Covariates

Covariates were measured at each exam. A binary indicator of current cigarette smoking was determined by self-report, defined as smoking regularly in the year prior to the examination (yes/no). Alcohol consumption was evaluated by self-reported average number of alcoholic drinks (e.g. beer, wine, cocktails) per week. Body mass index was calculated as the weight in kilograms divided by the square of the height in meters (kg/m^2^). Current antihypertensive medication use was self-reported and modeled as a binary variable (yes/no). "Baseline age" represented age at Examination 1. "Time from baseline age" was calculated as the difference between age at a given examination and the baseline age.

### Statistical Analyses

Primary analyses focused on associations of education (categorized as ≤12, 13-16, and ≥17 years, as described above) with longitudinal trajectories of SBP and DBP. Analyses relied on multivariable mixed linear models, which extend multiple linear regression to longitudinal analyses of repeated measures [11]. Accordingly, all effects reported in this study are likelihood-based estimates from mixed models, as are all 95% confidence intervals and test statistics used for inference about these estimates. The blood pressure measures from consecutive examinations represented repeated values of the continuous dependent variable. To model the dependence between repeated outcome measures, we used the autoregressive order 1 AR(1) covariance structure of the residuals, which assumed that blood pressure values measured at consecutive visits are correlated more strongly than those separated by longer time intervals [12]. All models adjusted for baseline age and time since baseline assessment (the former allowed us to adjust for potential cohort effects, such as increasing education over time in the United States). In further analyses, we additionally adjusted for several time-varying conventional risk factors for hypertension expected to be involved in potential mechanisms by which educational attainment may influence blood pressure (representing visit-specific binary indicators of current use of anti-hypertensive medication and current smoking, as well as time-varying continuous measures of alcohol consumption and body mass index). AR(1) is a standard choice for the covariance matrix in mixed model analyses of longitudinal data. Because there are no well established tests to compare fit of models' based on alternative covariance structures, the AR(1) structure is usually selected *a priori*. Assessment of the consistency of the AR(1) assumption is shown in the Results section. We implemented this longitudinal analysis by using PROC MIXED, with AR(1) covariance structure specified in REPEATED statement in SAS [13].

The second class of models additionally adjusted all the education effects for baseline blood pressure. Accordingly, in these models, baseline blood pressure values were *not *used as the outcome measure, so that the number of dependent value measures for each subject was reduced by one. Adjustment for baseline blood pressure effectively implied that we compared post-baseline trajectories of blood pressure as if participants with different education had the *same *baseline blood pressure. This approach allowed us to separate the antecedent effects of education, and other related factors, that might have resulted in the baseline differences in blood pressure, from the potential long-term effects of education on post-baseline changes in blood pressure.

Preliminary analyses were carried out to determine the most accurate representation of the effects of baseline age, and time since baseline. In particular, we *a priori *expected that both between- and within-subject effects of aging on blood pressure may be non-linear. Accordingly, we gradually expanded the basic model, with linear effects of age and time, by adding and testing first quadratic and then cubic effects of each of the two variables, while adjusting for the use of anti-hypertensive medication and for education. Furthermore, because we expected that the impact of within-subject aging (time) on blood pressure may vary depending on the baseline age, we also tested linear and quadratic interactions between age and time. All the multivariable mixed models employed in the final analyses, described below, adjusted for only those non-linear effects of age or time, and those interactions between these effects, that were statistically significant, based on Wald test with 2-tailed α = 0.05. Once the optimal representations of the effects of age and time, as well as of their interactions, were determined, these representations were used in the final analyses of the adjusted association of education with SBP and DBP. The final representation of the effects of baseline age and time from baseline for analyses on SBP was as follows: age+age^2^+time+time^2^+age*time+age*time^2^+age^2^*time. For DBP, it was as follows: age+age^2^+time+ time^2^+age*time+age*time^2^+age^2^*time+ age^2^*time^2^. All analyses were sex-specific, as a formal test for sex-by-education interaction suggested that the effects of education may differ between males and females (p = 0.046 for SBP; p = 0.063 for DBP).

## Results

Participants included in the current analyses had higher mean age (36.7 vs. 34.5 years, P < 0.001), and lower smoking rates (43.1% vs. 49.8%, P < 0.001) than excluded participants. Included and excluded participants had similar distributions of sex and baseline values of SBP, DBP, body mass index, alcohol consumption and antihypertensive medication use.

In females, unadjusted analyses demonstrated that education was inversely associated with baseline values of age, SBP, DBP, anti-hypertensive medication use, body mass index and smoking, and directly associated with alcohol consumption (Table 1). In males, education was inversely associated with age, SBP, DBP, body mass index, alcohol consumption and smoking.

**Table 1:** Baseline characteristics (means and proportion) of the Framingham Heart Study Offspring Cohort, according to educational attainment. Statistical variance shown in parentheses represents 95% confidence intervals.

		Educational Attainment (years)			
			
	All Participants	≤12	13-16	≥17	P*
**Female**					
N	2024	970	838	216	
Age (years)	36.2 (35.8, 36.6)	38.3 (37.7, 38.9)	34.8 (34.1, 35.4)	32.3 (31.2, 33.5)	<0.0001
Year of Birth	1937 (1936, 1937)	1935 (1934, 1935)	1938 (1938, 1939)	1941 (1940, 1942)	<0.0001
Systolic Blood Pressure, mmHg	118 (117, 119)	120 (119, 121)	117 (116, 118)	114 (112, 116)	<0.0001
Diastolic Blood Pressure, mmHg	75.9 (75.5, 76.3)	76.8 (76.1, 77.5)	75.7 (75.0, 76.3)	72.9 (71.7, 74.1)	<0.0001
Anti-Hypertensive Medication, %	3.0^†^	3.8	2.5	1.4	0.035
Alcohol Consumption (drinks/week)	4.4 (4.2, 4.7)	4.2 (3.8, 4.6)	4.5 (4.1, 4.9)	5.2 (4.2, 6.1)	0.037
Body Mass Index, kg/m^2^	24.0 (23.8, 24.1)	24.5 (24.3, 24.8)	23.5 (23.2, 23.7)	23.2 (22.7, 23.7)	<0.0001
Current Smoker, %	42.6^†^	47.3	39.3	33.8	<0.0001
**Male**					
N	1866	757	697	412	
Age (years)	37.2 (36.8, 37.7)	40.0 (39.3, 40.7)	35.6 (34.9, 36.3)	34.8 (33.9, 35.7)	<0.0001
Year of Birth	1936 (1935, 1936)	1933 (1932, 1934)	1937 (193, 1938)	1938 (1937, 1939)	<0.0001
Systolic Blood Pressure, mmHg	126 (126, 127)	128 (127, 129)	126 (125, 127)	124 (123, 125)	<0.0001
Diastolic Blood Pressure, mmHg	81.9 (81.5, 82.4)	82.9 (82.2, 83.7)	81.5 (80.7, 82.2)	80.8 (79.9, 81.7)	0.0008
Anti-Hypertensive Medication, %	3.5^†^	4.0	2.9	3.4	0.482
Alcohol Consumption (drinks/week)	11.0 (10.5, 11.6)	12.6 (11.6, 13.7)	10.5 (9.6, 11.3)	9.1 (8.1, 10.1)	<0.0001
Body Mass Index, kg/m^2^	26.5 (26.4, 26.7)	26.9 (26.6, 27.1)	26.4 (26.2, 26.7)	26.1 (25.7, 26.4)	0.0002
Current Smoker, %	43.7^†^	49.4	47.7	26.3	<0.0001

To test the trend across education level, we used t tests for continuous variables, and Cochran-Armitage tests for categorical variables.

^†^Pearson chi-square tests comparing males and females for proportion taking anti-hypertensive medication, and proportion current smokers demonstrated P-values of 0.41 and 0.49, respectively.

**Figure 1:**
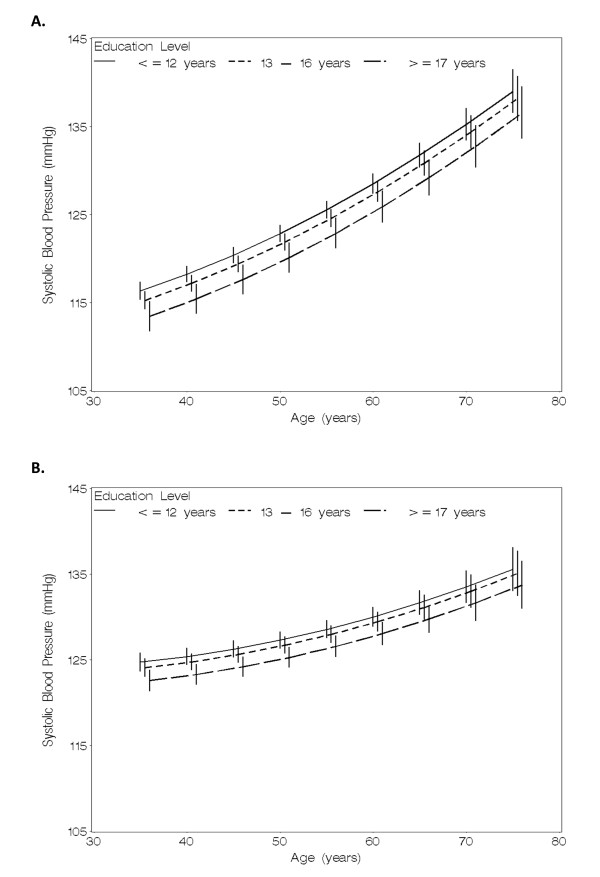
**Mixed linear models adjusted for age, demonstrating associations of educational attainment with longitudinal trajectories of mean systolic blood pressure (SBP) in (A) females and (B) males**. Age adjustment refers to adjustment for baseline age and time from baseline age. Modeling for baseline age and time from baseline was as follows: age+age^2^+time+time^2^+ age*time+age*time^2^+age^2^*time. Error bars represent 95% confidence intervals. Framingham Offspring Study, 1971-2001.

Using multivariable mixed linear models, mean SBP across the assessment times was higher for participants with low education compared with high education (Table 2, Figure 1), after adjusting for baseline age and time from baseline age, including their selected quadratic effects and two-way interactions, as shown in footnotes of Table 2. Specifically, in these analyses, the mean difference across all 7 visits in SBP for ≤12 *vs*. ≥17 years education was 3.26 (95% CI: 1.46, 5.05) mmHg in females, and 2.26 (95% CI: 0.87, 3.66) mmHg in males (Table 2). Further adjustment for conventional time-dependent covariates representing current (up-dated) values of antihypertensive medication use, smoking, body mass index and alcohol consumption reduced the difference in females to 2.86 (95% CI: 1.13, 4.59) mmHg, and in males to 1.25 (95% CI: -0.16, 2.66) mmHg.

**Table 2:** Multivariable-adjusted mixed linear models, demonstrating associations between educational attainment and longitudinal trajectories of mean systolic blood pressure, Framingham Offspring Study, 1971-2001.

		**Model Adjustment**			
		
**Sex (n)**	**Education (Years)**	**Age**	**Age, Conventional Risk Factors**	**Age, Baseline Blood Pressure**	**Age, Baseline Blood Pressure, Conventional Risk Factors**
	
Female (n = 2024)	≤12	**3.26 (1.46, 5.05)**	**2.86 (1.13, 4.59)**	**2.69 (1.09, 4.30)**	**2.53 (0.93, 4.14)**
	13-16	**2.00 (0.20, 3.79)**	**2.14 (0.42, 3.87)**	1.30 (-0.31, 2.91)	1.47 (-0.12, 3.07)
	≥17	0	0	0	0
Male (n = 1866)	≤12	**2.26 (0.87, 3.66)**	1.25 (-0.16, 2.66)	1.20 (-0.07, 2.46)	0.34 (-0.97, 1.64)
	13-16	**1.55 (0.16, 2.94)**	0.88 (-0.51, 2.27)	0.98 (-0.28, 2.24)	0.39 (-0.90, 1.68)
	≥17	0	0	0	0

Point estimates (and 95% confidence intervals shown in parentheses) represent mean differences in systolic blood pressure (mmHg) between comparison and referent groups. Age adjustment refers to adjustment for baseline age and time from baseline age. Modeling for baseline age and time from baseline was as follows: age+age^2^+time+ time^2^+age*time+age*time^2^+age^2^*time.

Conventional risk factors include antihypertensive medication, smoking, body mass index and alcohol consumption.

A second set of analyses adjusted for baseline SBP, in an effort to evaluate if there were educational differences in the post-baseline values of blood pressure, independent of the baseline differences. In analyses adjusted for baseline age, time from baseline age, and baseline SBP, females with ≤12 years education had 2.69 (95% CI: 1.09, 4.30) mmHg higher SBP over follow-up compared with females with ≥17 years education (Table 2). Further adjustment for conventional risk factors had minimal impact on the effect strength in females (effect reduced to 2.53 (95% CI: 0.93, 4.14) mmHg for ≤12 years vs. ≥17 years education). Associations were weaker in males, where those with ≤12 years education had 1.20 (95% CI: -0.07, 2.46) mmHg higher SBP over follow-up compared to males with ≥17 years of education, after adjustment for baseline age, time from baseline age, and blood pressure; effects were substantially reduced after adjusting for conventional risk factors (Table 2). As a formal test for sex-by-education interaction suggested that the effects of education may differ between males and females (p = 0.046 for SBP), and of the covariates only alcohol consumption showed differential associations with education between males and females (Table 1), analyses were repeated without adjusting for alcohol consumption. This approach evaluated if gender differences in associations between education and SBP persisted with and without adjusting for alcohol consumption. Analyses adjusted for all aforementioned covariates with the exception of alcohol (i.e. adjusted for baseline age, time from baseline age, baseline SBP, antihypertensive medication, smoking, and body mass index) demonstrated persistent gender differences in associations where mean difference across all 7 visits in SBP for ≤12 *vs*. ≥17 years education was 2.20 (95% CI: 0.59, 3.81) mmHg in females, and 0.60 (95% CI: -0.72, 1.92) mmHg in males, suggesting gender differences in the association consumption between alcohol and education were not a substantial explanation for gender differences in observed associations between education and SBP.

DBP was higher in female, but less so in male, study participants of low compared to high educational attainment after adjusting for baseline age and time since baseline assessment including the selected quadratic effects and two-way interactions described in Table 3. Specifically, the mean difference in DBP across all assessment times, for ≤12 *vs*. ≥17 years education was 1.47 (95% CI: 0.43, 2.50) mmHg in females, and 0.66 (95% CI: -0.17, 1.50) mmHg in males (Table 3). Further adjustment for conventional risk factors including antihypertensive medication, smoking, body mass index and alcohol consumption reduced the association strength of low education with DBP somewhat in females, resulting in a smaller difference of 1.26 (95% CI: 0.25, 2.26) mmHg, and eliminated any association in males, with adjusted difference of 0.05 (95% CI: -0.78, 0.86) mmHg. In analyses adjusted for baseline age, time from baseline age, and baseline SBP, there was no association between education and post-baseline values of DBP, among participants with the same baseline DBP, in either sex (Table 3).

**Table 3:** Multivariable-adjusted mixed linear models, demonstrating associations between educational attainment and longitudinal trajectories of mean diastolic blood pressure, Framingham Offspring Study, 1971-2001.

		Model Adjustment			
		
Sex (n)	Education (Years)	Age	Age, Conventional Risk Factors	Age, Baseline Blood Pressure	Age, Baseline Blood Pressure, Conventional Risk Factors
Female (n = 2024)	≤12	**1.47 (0.43, 2.50)**	**1.26 (0.25, 2.26)**	0.62 (-0.31, 1.55)	0.51 (-0.42, 1.43)
	13-16	**1.29 (0.26, 2.33)**	**1.40 (0.40, 2.40)**	0.33 (-0.60, 1.26)	0.44 (-0.48, 1.36)
	≥17	0	0	0	0
Male (n = 1866)	≤12	0.66 (-0.17, 1.50)	0.05 (-0.78, 0.86)	0.42 (-0.33, 1.18)	-0.09 (-0.85, 0.68)
	13-16	0.60 (-0.23, 1.43)	0.14 (-0.68, 0.96)	0.49 (-0.27, 1.24)	0.09 (-0.67, 0.85)
	≥17	0	0	0	0

Point estimates (and 95% confidence intervals shown in parentheses) represent mean differences in diastolic blood pressure (mmHg) between comparison and referent groups. Age adjustment refers to adjustment for baseline age and time from baseline age. Modeling for baseline age and time from baseline was as follows: age+age^2^+time+ time^2^+age*time+age*time^2^+age^2^*time+age^2^*time^2^.

Conventional risk factors include antihypertensive medication, smoking, body mass index and alcohol consumption.

In order to assess the consistency of the AR(1) assumption with our data, we estimated the Pearson correlation coefficients between measurements at different time points, for both SBP and DBP, separately for female and male. The results of Pearson correlation coefficients (shown in Tables 4, 5, 6, 7) indeed show an autoregressive correlation structure, that is, the correlation decreases systematically as the distance in time between two measurements increases. In addition, we compared the AIC of the AR(1) model with that based on another popular structure: the exchangeable structure. As expected, based on Tables 4, 5, 6, 7 and *a priori *considerations, the AR(1) (e.g. for male in the model specified in the last column of Table 2, AIC = 73281) yielded better (i.e. lower AIC) than the exchangeable structure (AIC = 73434).

**Table 4:** Pearson correlation coefficients of systolic blood pressure among females for examinations 1-7.

	Exam 1	Exam 2	Exam 3	Exam 4	Exam 5	Exam 6	Exam 7
Exam 1	1.000	0.620	0.590	0.531	0.492	0.441	0.370
Exam 2	0.620	1.000	0.717	0.660	0.600	0.556	0.465
Exam 3	0.590	0.717	1.000	0.744	0.671	0.629	0.526
Exam 4	0.531	0.660	0.744	1.000	0.707	0.637	0.546
Exam 5	0.492	0.600	0.671	0.707	1.000	0.646	0.572
Exam 6	0.441	0.556	0.629	0.637	0.646	1.000	0.669
Exam 7	0.370	0.465	0.526	0.546	0.572	0.669	1.000

**Table 5:** Pearson correlation coefficients of systolic blood pressure among males for examinations 1-7.

	Exam 1	Exam 2	Exam 3	Exam 4	Exam 5	Exam 6	Exam 7
Exam 1	1.000	0.554	0.513	0.461	0.406	0.330	0.303
Exam 2	0.554	1.000	0.681	0.592	0.495	0.462	0.418
Exam 3	0.513	0.681	1.000	0.679	0.588	0.538	0.460
Exam 4	0.461	0.592	0.679	1.000	0.661	0.567	0.452
Exam 5	0.406	0.495	0.588	0.661	1.000	0.592	0.516
Exam 6	0.330	0.462	0.538	0.567	0.592	1.000	0.584
Exam 7	0.303	0.418	0.460	0.452	0.516	0.584	1.000

**Table 6:** Pearson correlation coefficients of diastolic blood pressure among females for examinations 1-7.

	Exam 1	Exam 2	Exam 3	Exam 4	Exam 5	Exam 6	Exam 7
Exam 1	1.000	0.572	0.511	0.443	0.350	0.250	0.121
Exam 2	0.572	1.000	0.686	0.597	0.498	0.369	0.240
Exam 3	0.511	0.686	1.000	0.679	0.548	0.465	0.291
Exam 4	0.443	0.597	0.679	1.000	0.589	0.483	0.329
Exam 5	0.350	0.498	0.548	0.589	1.000	0.557	0.455
Exam 6	0.250	0.369	0.465	0.483	0.557	1.000	0.576
Exam 7	0.121	0.240	0.291	0.329	0.455	0.576	1.000

**Table 7:** Pearson correlation coefficients of diastolic blood pressure among males for examinations 1-7.

	Exam 1	Exam 2	Exam 3	Exam 4	Exam 5	Exam 6	Exam 7
Exam 1	1.000	0.533	0.453	0.373	0.273	0.158	0.061
Exam 2	0.533	1.000	0.625	0.498	0.406	0.292	0.202
Exam 3	0.453	0.625	1.000	0.593	0.489	0.375	0.322
Exam 4	0.373	0.498	0.593	1.000	0.555	0.447	0.337
Exam 5	0.273	0.406	0.489	0.555	1.000	0.520	0.447
Exam 6	0.158	0.292	0.375	0.447	0.520	1.000	0.566
Exam 7	0.061	0.202	0.322	0.337	0.447	0.566	1.000

## Discussion

Findings in this paper demonstrated that education was inversely associated with longitudinal trajectories of mean SBP in females and males. Furthermore, especially in females, lower education was associated with higher post-baseline SBP even among the participants with the same baseline SBP. This suggests that low education may have a long-term impact on changes over time in blood pressure in females. Adjusting for the time-varying values of conventional risk factors, measured at the same time as the blood pressure, typically reduced the strength of these associations. Associations of education with DBP were generally weaker than with SBP, for both females and males.

### Prior Literature

Few studies have investigated sex-specific longitudinal trajectories of blood pressure, particularly over a substantial proportion of the life course. Diez Roux *et al*. in the ARIC cohort (n = 8555) aged 45 to 64 years at baseline and followed using 4 examinations over a period of 9 years, found in white participants, that education was marginally inversely associated with increases in blood pressure after adjusting for age, sex, center, medication use, and reported interactions between time and sex, and interactions between time and baseline age [4]. The 5-year change in mean SBP was 6.0 mmHg for those with <high school degree and 5.3 mmHg for those with a college degree. Further adjustment for baseline SBP somewhat reduced the association strength, to 5.9 mmHg for <high school and 5.5 mmHg for participants with college degree. Associations were weaker in black participants. Strand et al. demonstrated, in a large prospective study of 48,422 males and females aged 35-49 followed for 14 years using three examinations, that education was inversely associated with increases over time in SBP in males and females, after adjusting for year of birth [6]; socioeconomic disparities widened over time in females but not males. In a study on the Framingham Offspring cohort that included only participants aged 20-29 years at baseline (many of whom may not have completed education yet), education was not significantly associated with mean 8-year change in SBP or DBP in males or females, after adjusting for age [5]. In the CARDIA study of 2913 participants aged 18-30 years at baseline education was significantly inversely associated with mean 15-year change in both SBP and DBP [7]. Specifically for SBP, those with <high school degree had a 15-year mean increase of 8.2 mmHg versus only 0.7 mmHg for participants with >college graduate degree. However the observed associations were not adjusted for covariates [7]. Although prior cross-sectional studies suggested that associations may be stronger in females than males [3], little is known about sex-specific associations between education and blood pressure trajectories, particularly over long periods of the life course (>20 years follow-up). Finally, little is known about the effects of adjusting for use of antihypertensive medications, body mass index, alcohol consumption, smoking or other potential mechanisms that may, at least partly, mediate the impact of lower education on longitudinal trajectories of blood pressure. This study added to the literature sex-specific information demonstrating that education is inversely associated with longitudinal trajectories of mean SBP in females and males over a substantial proportion of the life course (approximately 30 years) and that association may be stronger in females than males. Furthermore, in females, lower education was associated with a higher mean post-baseline SBP even among participants with the same baseline SBP, suggesting a possible long-term impact of lower education. Adjusting for up-dated values of conventional risk factors typically reduced strengths of association, but in females the impact of lower education remained statistically significant. For DBP, association strengths were generally weaker for both females and males.

### Mechanisms

The primary candidate mechanisms by which education may influence longitudinal trajectories of blood pressure involve conventional risk factors for hypertension, including smoking, obesity, blood pressure medication use, and alcohol consumption. In this study, in females, education was inversely associated with anti-hypertensive medication use, body mass index and smoking, and directly associated with alcohol consumption. In males, education was inversely associated with body mass index, alcohol consumption and smoking, and not associated with antihypertensive use. Furthermore, the estimated effects of education tended to somewhat decrease after adjusting for these potential mechanisms (particularly in males), suggesting that they may be at least partial explanatory pathways for the observed association between educational attainment and longitudinal trajectories of blood pressure. It is important to note that biases can be induced by adjusting for variables that may partly mediate the effect of exposure; therefore, these mechanistic findings should be interpreted with caution [14]. Furthermore, there remain plausible confounders unadjusted for, such as childhood socioeconomic circumstances (which are associated with adulthood education and blood pressure [15]), parental blood pressure (which may be associated with offspring education and has been related to offspring blood pressure [16]), intelligence (which is associated with educational attainment and CHD risk [17]), and early life obesity (that could affect upward social mobility via obesity discrimination particularly in women [18,19], and is related to blood pressure in adulthood [20]. Consequently, residual confounding remains a possibility.

Low educational attainment has been demonstrated to predispose individuals to high strain jobs, characterized by high levels of demand and low levels of control, which have been associated with elevated blood pressure [21,22]. Other related mechanisms involve stress-induced sympathetic nervous system activation due to stressful conditions outside of work, that are also associated with low educational attainment. These may be particularly important for women. It has been shown that women with low education have higher risk of co-occurring psychosocial determinants of poor health, including single-parenting, depression, income below the poverty threshold, and unemployment, compared to men with low education [23]. Consequently, low socioeconomic position may be a stronger determinant of hypertension risk in women compared with men. This may be one of the explanations for why we found a significant interaction between sex and education, and somewhat stronger associations between education and blood pressure in women than men. The extent of health care available for people of low socioeconomic position is typically less than what is available for those with high socioeconomic position, hence limiting access to treatments of hypertension [24]. Furthermore, there is evidence that people of low socioeconomic position have less healthful diets, such as lower rates of fruit and vegetable consumption, and higher salt intake, which may be additional mechanisms contributing to disparities in blood pressure [25,26].

It has been demonstrated that although both SBP and DBP are positively associated with incidence of coronary heart disease, there are differences in the way SBP and DBP evolve over the life course. SBP tends to increase steadily with age, while DBP tends to increase until age 50 years, and to decrease steadily after that age [4,8,27]. The mechanisms responsible for the age-related increase in DBP among younger people likely involve an atherosclerotic increase in peripheral resistance, caused by narrowing of the smaller arteries and arterioles [8,28]. In contrast, for older individuals, structural damage and calcification due to arteriosclerosis in the larger conduit arteries can result in loss of arterial compliance, which can cause a rise in SBP, but a reduction in DBP [8,28]. As the burden of hypertension is greatest after the age of 50 years, and it is exceedingly uncommon to have diastolic hypertension without concurrent systolic hypertension in adults over the age of 50 years, it has been argued that SBP is by far the more important measure of the two in terms of predictive importance for population health [8].

Studies generally show consistent inverse associations between educational attainment and longitudinal changes in SBP [4,6,7], with the exception of young participants aged 20-29 years at baseline, followed over 8 years in the study by Hubert *et al*. [5] However, findings are less consistent for DBP, where studies have shown inverse [7], null [5], or even positive [4] associations between educational attainment and longitudinal changes in DBP. Our findings demonstrated fairly robust inverse associations of education with SBP, and weaker inconsistent associations with DBP. The pathophysiological mechanisms (e.g. smoking, obesity, alcohol consumption) that cause steady increases over the life course for SBP but not DBP, and also tend to be inversely associated with socioeconomic position, may explain the more consistent findings for the inverse association between education and changes in SBP rather than DBP over the life course. However, adjustment for these variables in our study appeared to account for only a small amount of the association in females, and a larger amount of the (weaker) association in males, suggesting there may be other explanatory factors, particularly in females.

### Strengths and Weaknesses

Strengths of this study include having access to data on approximately 30 years of longitudinal blood pressure measurements. Furthermore, follow-up rates of the Framingham Heart Study are considered to be high for observational studies, decreasing risk of bias due to loss-to-follow-up. Finally, measurements of blood pressure were performed using methods and equipment providing good accuracy and precision, and analyses relied on statistical methods appropriate for longitudinal repeated-measures studies.

With regard to weaknesses, because the historical design of the Framingham Offspring Study reflected the population of Framingham, Massachusetts at study onset in 1948, the Original and Offspring cohorts are largely composed of white participants. Consequently, the generalizability of our findings to other races/ethnicities is uncertain. Furthermore, although we had up to 7 measurements for each covariate, we expect there to be reasonable residual confounding due to imperfect measurement of obesity (body mass index), and self-reported alcohol consumption, smoking and antihypertensive medication use.

## Conclusion

This study provides evidence that education is inversely associated with systolic blood pressure throughout a 30 year life course span, and associations may be stronger in females than males. These findings provide evidence that education may be a potential risk factor for elevated blood pressure across the life course.

## Competing interests

The authors declare that they have no competing interests.

## Authors' contributions

EL initially conceived of the study and further developed the study objectives in collaboration with all co-authors. EL wrote much of the initial draft of the manuscript. MA was the senior biostatistics advisor and drafted the analytic approach in the Methods section. YX performed the analyses and advised on the analytic approach. JL made substantial contributions to the analytic approach, and advised on the subject matter related to socioeconomic disparities in blood pressure. All authors were involved with drafting the final manuscript, and revising it as needed for important intellectual content. All authors read and approved the final manuscript.

## Pre-publication history

The pre-publication history for this paper can be accessed here:

http://www.biomedcentral.com/1471-2458/11/139/prepub
